# Reduction of in utero lead exposures in South African populations: Positive impact of unleaded petrol

**DOI:** 10.1371/journal.pone.0186445

**Published:** 2017-10-16

**Authors:** Halina B. Röllin, Bukola Olutola, Kalavati Channa, Jon Ø. Odland

**Affiliations:** 1 School of Health Systems and Public Health, Faculty of Health Sciences, University of Pretoria, Pretoria, South Africa; 2 Environment and Health Research Unit, Medical Research Council, Johannesburg, South Africa; 3 Lancet Laboratories, Department of Analytical Chemistry, Johannesburg, South Africa; 4 Department of Biomedical Technology, School of Health Sciences, University of Johannesburg, Johannesburg, South Africa; 5 Institute of Community Medicine, University of Tromsø, Tromsø, Norway; Ludwig-Maximilians-Universitat Munchen, GERMANY

## Abstract

**Background:**

Prenatal exposure to lead (Pb) has been shown to have negative and irreversible health impacts on foetal and early childhood development, affecting morbidity and mortality in adulthood. This study aimed to assess *in utero* Pb exposure, examine birth outcomes, and identify confounding factors in the large cohort of South African population, following the legislated removal of Pb from petrol.

**Methods:**

Lead was measured in the maternal blood, urine and cord blood using Inductive Coupled Plasma Mass spectrometry (ICP-MS). The statistical analyses included Spearman’s correlation, Wilcoxon rank sum (Mann Whitney), Kruskal-Wallis rank tests and multivariate linear regression.

**Results:**

Overall, the geometric mean (GM) of Pb in maternal blood (PbB) was 1.32 μg/dL (n = 640; 95% CI, 1.24–1.40). In the subset cohort, the GM of paired maternal PbB and cord blood (PbC) was 1.73 μg/dL (n = 350; 95% CI, 1.60–1.86) and 1.26 μg/dL (n = 317; 95% CI, 1.18–1.35), respectively with a positive correlation between the log PbB and the log PbC (rho = 0.65, p = <0.001). Birth outcomes showed geographical differences in the gestational age (p<0.001), birth length (p = 0.028) and head circumference (p<0.001), Apgar score at 5 min (p<0.001) and parity (p<0.002). In female neonates, a positive association was found between PbC and head circumference (rho = 0.243; p<0.016). The maternal PbB levels were positively correlated with race, educational status, water sources, cooking fuels and use of pesticides at home.

**Conclusions:**

This study has demonstrated not only the positive impact that the introduction of unleaded petrol and lead-free paint has had on *in utero* exposure to Pb in South Africa, but has also contributed new data on the topic, in a region where such data and scientific investigations in this field are lacking. Future research should evaluate if similar effects can be detected in young children and the adult population.

## Introduction

The detrimental effects of lead (Pb) on human development and growth are well recognised, and remain an issue of concern for public health. Even at low levels of Pb exposure, neurotoxic effects such as reduced intelligence quotient (IQ), hyperactivity, poor school performance and achievement, and violent behaviours in childhood and adolescence are evident [1,2]. Many studies have shown and confirmed that there are no safe levels of Pb exposure in humans, particularly in susceptible populations such as pregnant women and their developing foetuses [3]. During pregnancy, Pb has the ability to cross the placenta, as well as the blood-brain-barrier (BBB), resulting not only in neurotoxicity to the developing foetus, but also negatively impacting on early childhood development [4,5]. The physiological and pathological changes during pregnancy may also cause mobilisation of Pb from maternal bone tissue (95% of the adult body burden of Pb is stored in bone) [6,7]. Studies have shown that a decrease in calcium intake during pregnancy causes an increase in maternal blood Pb (PbB) concentrations, as a direct effect of mobilization of Pb from bone. This results in foetal uptake of Pb which continues after birth and through lactation [8, 9]. Elevated PbB levels in pregnant women have been shown to affect birth outcomes by reducing foetal growth, increasing rates of congenital anomalies and promoting premature labour, although these studies are not yet conclusive [10–12].

Thus, sources of environmental Pb are well identified and attempts to reduce exposure levels worldwide remains a priority; however, the persistence of Pb is an obstacle, particularly in poor resourced countries [13]. Historical sources of Pb include leaded gasoline/petrol and lead-based paints [14]. Since the removal of Pb as an anti-knocking additive in gasoline in developed countries, PbB levels in both general and susceptible populations have declined exponentially over time, and the prevalence of children with PbB levels exceeding the 10 μg/dL concentration of concern has dropped considerably [15,16]. This accomplishment in terms of benefitting public health has been brought about by highly effective control measures set out by the respective governments, combined with education and public awareness initiatives by the public health sector [17].

In developing countries, Pb toxicity in vulnerable groups remains problematic as no mitigation, Pb surveillance or screening programmes are in place. In many countries, including South Africa, there is historical use of Pb in petrol, and Pb from other sources such as mining, refining, and industrial applications [18]. In addition, due to the depressed economy, more and more people including pregnant women, participate in the cottage and informal industries that may unknowingly expose them to Pb [19]. In these vulnerable populations, there are confounding factors for Pb toxicity, including poor health status, compromised diet and substandard living conditions.

Due to technical and financial reasons, the removal of Pb from petrol has taken a longer time in developing countries. For example, South Africa embarked on a gradual phasing out of Pb from petrol in1986, but final elimination was achieved only twenty years later, in January 2006 [14, 20]. Furthermore, it was only in 2009 that the use of Pb in household paints, children’s toys and other everyday goods was banned by legislation [21].

A number of studies performed in Africa and South Africa have shown that exposure to Pb is prevalent, especially in young children [22–25]. Two studies from South Africa have demonstrated that the introduction of unleaded petrol in 2006 correlated directly with decreased PbB levels among school children [26, 27].

To date, only a few South African studies have investigated prenatal Pb exposure. The “Birth to Twenty” longitudinal study on a cohort of children born in 1990 in Soweto, Johannesburg, showed that 52% of neonates exceeded PbB levels of 5μg/dL [28]. Another study performed in 1996 in the KwaZulu Natal Indian Ocean coastal city of Durban, PbB levels in maternal and umbilical cord blood (hereafter referred to as cord blood) were found to be above 10 μg/dL in 18.7% of the women and in 12% of paired cord blood samples [18]. Our pilot study performed in 2006 (the year of final elimination of Pb from petrol) in various geographical regions of South Africa indicated significantly lower exposures and clear geographical differences in PbB levels in delivering women compared to previous studies [29, 30].

The primary aim of the current study was to assess Pb exposures *in utero* and the possible effects of this exposure on birth outcomes in a large cohort of populations residing along coastal regions of South Africa. Additional aims were to identify contributing factors to Pb exposure and to evaluate if the removal of Pb from South African petrol in 2006 impacted on *in utero* exposures to Pb, in the study population.

## Materials and methods

### Study sites and study population

**Fig 1:**
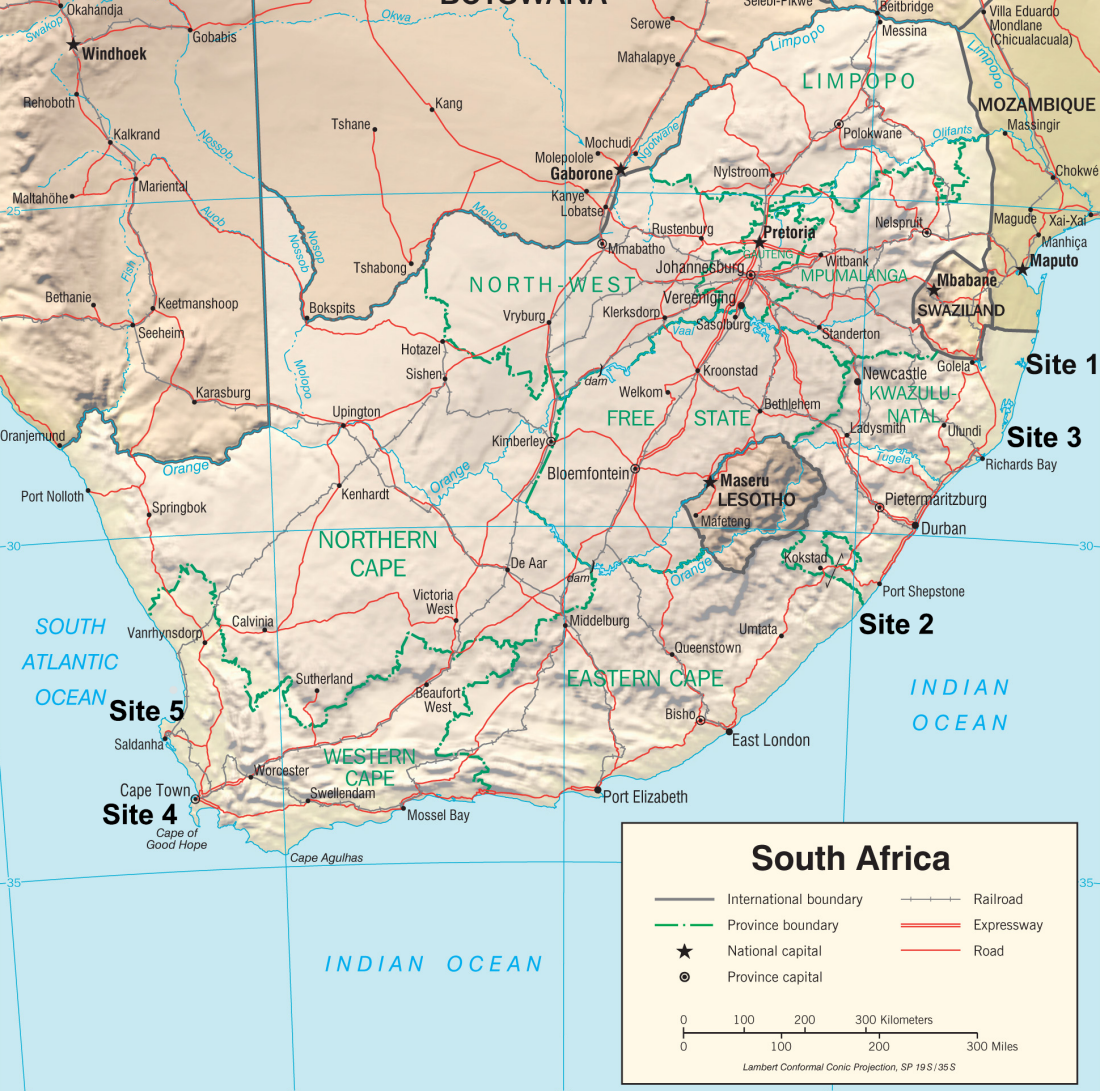
Study sites: Sites 1, 2, 3—Indian Ocean; sites 4 and 5—Atlantic Ocean. **Figure is identical but sites locations were added, and is therefore for representative purposes only.** (https://www.cia.gov/library/publications/resources/cia-maps-publications/map-downloads/South%20Africa_Physiography).

Five sites were included in the study: three sites (Sites 1 to 3) were situated in the KwaZulu Natal Province along the Indian Ocean coast (sample collection took place in 2008), and two sites (Sites 4 and 5) were situated in the Western Cape Province along the Atlantic Ocean coast (sample collection took place in 2012 and 2013, respectively (Fig 1). All study sites were rural, except for the urban site of the city of Cape Town (Site 4). The potential candidates recruited for the study were women who were admitted for delivery at the local maternity sections at public hospitals. Women who agreed to participate in the study signed an informed consent form and agreed to donate blood and urine samples before delivery, and to the collection of cord blood samples after delivery. Participants agreed to answer a socio-demographic questionnaire which also included the topics of diet, lifestyle, and self-reported health status. The dietary part of the questionnaire recorded the frequency of intake of various basic foods during pregnancy. Participants also consented to access and use of hospital birth outcome data (including maternal and neonate characteristics such as weight, length and head circumference, gestational age, Apgar score, as well as birth complications, if any) for research purposes. In total, 650 women answered the questionnaire and 640 donated pre-partum blood samples. In a subset cohort of 350 women (from the Indian Ocean site), urine and cord blood samples (n = 317) were also collected.

### Sample collection

Ten mL of venous blood was collected before delivery into a BD Vacutainer tube (containing EDTA) and 10 mL of paired cord blood post-partum. Urine (30 mL) was collected before delivery. All samples were stored at -20°C and transported to the South African National Institute for Occupational Health (NIOH) laboratory for analysis. The NIOH participates in the Wadsworth Center–New York State Department of Health Proficiency testing scheme for whole blood and urine. The results obtained are consistently accepted with no indication of bias.

### Analytical procedures

The analyses for Pb in the paired maternal and cord whole blood and maternal urine were performed using an Inductively Coupled Plasma-Mass Spectrometer (ICP-MS) instrument (Agilent 7500ce ICP-MS with an Octopole Reaction System). Contamination-free vessels and procedures were used throughout, and validation of results was accomplished by including certified standards, as well as certified reference quality controls, in the analyses.

Digested blood samples were analysed for Pb (208) using the no gas acquisition mode and Tl (205) was used as an internal standard. For quality assurance, two certified blood reference controls, viz. Seronorm ^TM^ Trace Elements (Sero LTD., Billingstad, Norway) (Levels 1 and 2) were analysed with every analytical run, at intervals between every 10 samples. The detection limit (three times the standard deviation of all blank samples) for Pb in whole blood was 0.04 μg/dL.

Acidified urine samples were analysed using the no gas acquisition mode and Au (197) as an internal standard. For urine, five certified reference controls were analysed with every analytical run in intervals of 12 samples (Seronorm^TM^ Trace Elements in urine (urine blank), Lot: OK4636; Seronorm^TM^ Trace Elements in urine, Lot: 0511545; UTAK Urine control, Lot: 1170; Lyphochek Urine Metals Control level 1, Lot: 69121; Lyphochek Urine Metals Control level 2, Lot: 69122). The detection limit (three times the standard deviation of all blank samples) for Pb in urine was 0.52 μg/L.

### Covariates

Covariate information was obtained during the questionnaire-based interview and from medical records. Maternal weight and height were recorded at the hospital on admission. From the medical records, the following neonate characteristics were retrieved: birth weight (grams), birth length (cm), head circumference (cm) and gestational age (weeks). Pre-term labour was defined as mothers giving birth at less than 37 weeks gestational age. Education was categorised as no education to completed primary school, completed secondary school and any level of tertiary education reached. Maternal tobacco smoking during pregnancy was defined as yes or no. Exposure to environmental tobacco smoke (ETS) was defined as exposure to tobacco smoke from smoking by others in the household. A binary classification was used for exposure to indoor smoke from the burning of fossil fuel (wood and coal) for the purpose of heating or cooking, separating study participants into those exposed to fossil fuel and those not exposed (for example, those using electricity). Dietary questions relating to the intake of proteins, carbohydrates, dairy products, tea, coffee, bottled water, fruits, as well as vine, root and leafy vegetables, were assessed and classified as daily, at least once a week and seldom (both for pre-pregnancy and during pregnancy).

### Statistical analyses

The statistical analyses were performed using STATA (StataCorp, 2013. Stata Statistical Software: Release 13. College Station, TX: StataCorp LP). Pb was detected in all blood and urine samples. Bivariate analyses between maternal PbB exposure and covariates were evaluated by Spearman’s correlation coefficient, Wilcoxon rank sum (Mann Whitney), Kruskal-Wallis rank tests and linear regression as appropriate. The distribution of Pb levels in maternal blood and in cord blood, were skewed and were log transformed. Multivariate linear regression was carried out using a backward deletion approach, starting with a full model of factors significantly associated with natural logarithm-transformed maternal Pb levels in the bivariate analysis. All statistical tests were two tailed and statistical significance was set at p < 0.05.

### Ethical considerations

Ethics approval for the study was obtained from the Human Research Ethics Committee of the University of Witwatersrand in Johannesburg (Protocol no. M10742), and from the relevant provincial Departments of Health: the Provincial Department of Health of KwaZulu Natal and the Western Cape Provincial Department of Health. In addition, CEOs of the respective hospitals had to confirm that he/she allowed the research work to proceed. Identical procedures were followed in terms of obtaining consent from participants. Confidentiality was maintained by assigning identification numbers to all study participants. During the informed consent process, it was emphasised that participation was voluntary and could be withdrawn at any time.

## Results

### Characteristics of the study population

The most important population characteristics specified by total cohort and geographic position (namely the Indian and Atlantic Ocean) are presented in Table 1.

**Table 1:** Population characteristics for the total cohort, and by geographical position.

Characteristic	Total % (95% CI*)	Indian Ocean % (95% CI*)	Atlantic Ocean % (95% CI*)	p-value
**Mothers characteristics**				
Age (years)	25.2 (24.7–25.7)	24.5 (23.8–25.2)	26.1 (25.4–26.7)	<0.001
**Marital status**				
Married	22.9 (20.0–26.1)	12.0 (8.5–15.5)	35.4 (30.0–40.8)	
Single/Divorced/widowed	59.6 (55.8–63.5)	73.4 (68.7–78.1)	43.8 (38.1–49.5)	
Co-habiting	17.5 (14.6–20.5)	14.6 (10.9–18.4)	20.9 (16.2–25.5)	<0.001
**Education**				
None/Primary	8.6 (6.4–10.9)	15.2 (11.3–19.2)	1.4 (0.0–2.7)	
Secondary	64.5 (60.7–68.2)	47.0 (41.5–52.4)	83.8 (79.6–88.1)	
Tertiary	26.9 (23.4–30.4	37.8 (32.5–43.1)	14.8 (10.8–18.9)	<0.001
**Percentage unemployed**	75.9 (72.6–79.3)	86.0 (82.3–89.7)	64.3 (58.8–69.8)	<0.001
**Ownership of home**				
Owned	83.1 (80.2–86.0)	94.2 (91.7–96.7)	70.2 (64.9–75.4)	
Rented	16.9 (14.0–19.8)	5.8 (3.3–8.3)	29.8 (24.6–35.1)	<0.001
**Housing type**				
Formal housing	74.0 (70.6–77.4)	83.3 (79.4–87.3)	63.2 (57.7–68.7)	
Flat	5.2 (3.5–6.9)	3.5 (1.6–5.5)	7.1 (4.2–10.0)	
Backyard dwelling	4.2 (2.7–5.8)	0.9 (-0.0–1.9)	8.1 (5.0–11.2)	
Informal housing	13.2 (10.5–15.8)	7.6 (4.8–10.4)	19.6 (15.1–24.1)	
Other	3.5 (2.0–4.9)	4.7 (2.4–6.9)	2.0 (0.4–3.6)	<0.001
**Residence**				
Urban/Informal settlement	14.0 (11.2–16.7)	5.0 (2.7–7.3)	25.5 (20.2–30.7)	
Rural	52.4 (48.4–56.4)	90.4 (87.2–93.5)	3.8 (1.5–6.0)	
Peri urban	33.7 (30.0–37.4)	4.7 (2.4–6.9)	70.8 (65.3–76.3)	<0.001
**Race/ethnicity**				
African Black	78.4 (75.2–81.6)	98.8 (97.7–100.0)	54.6 (48.9–60.3)	
Other	21.6 (18.4–24.8)	1.2 (0.0–2.3)	45.4 (39.7–51.1)	<0.001
**Fuel used for cooking**				
Electricity	74.8 (71.5–78.2)	56.7 (51.4–62.0)	96.0 (93.7–98.2)	
Paraffin	5.3 (3.6–7.1)	7.6 (4.8–10.4)	2.7 (0.8–4.6)	
Gas/wood	19.8 (16.8–23.0)	35.8 (30.7–40.9)	1.4 (0.03–2.7)	<0.001
**Source of drinking water**				
Indoor tap	38.3 (34.5–42.1)	10.7 (7.4–14.0)	69.7 (64.4–75.0)	
Outdoor tap	52.1 (48.2–56.0)	71.2 (66.4–76.1)	30.3 (25.1–35.6)	
Borehole	5.1 (3.3–6.8)	9.5 (6.4–12.6)	0	
River/stream	4.6 (2.9–6.2)	8.6 (5.6–11.6)	0	<0.001
**Exposure to passive smoking in the household**				
No	59.8 (56.0–63.6)	67.9 (62.9–72.8)	50.5 (44.8–56.3)	
Yes	40.2 (36.4–44.1)	32.2 (27.2–37.2)	49.5 (43.8–55.2)	<0.001
**Perception that air quality is inferior in the neighbourhood**	24.8 (21.5–28.2)	10.8 (7.5–14.1)	41.1 (35.5–46.7)	<0.001
**Perception of environmental pollution around the home**	24.3 (21.0–27.7)	11.1 (7.7–14.4)	40.2 (34.5–45.9)	<0.001

*CI—confidence interval

Of the total study cohort (n = 650), 350 participants resided along the Indian Ocean coast, and 300 participants along the Atlantic Ocean coast of South Africa. Of the total study cohort (n = 650), 450 women (69%) resided in rural areas, and 200 women (31%) in an urban area. Ethnicity of most of the participants was African Black (78%), with 45% of the women residing along the Atlantic Ocean coast being Coloured. Women from the Indian Ocean sites were significantly younger than those from the Atlantic Ocean sites (p<0.001). Along the Indian Ocean sites, more than a third (37.8%) of the subjects had attained tertiary education level, compared to only 14.8% from the Atlantic Ocean sites. Collectively, the large majority of participants was unemployed, but with a significantly lower rate of unemployment at the Atlantic Ocean sites (p<0.001). The majority of the participants lived in formal housing (74%) owned by self or family members, but in the Atlantic Ocean sites, more than 19% of participants were living in informal dwellings (shacks).

In all of the study sites, electricity was the predominant source of energy for cooking, The majority of rural women from the Indian Ocean sites reported sourcing their potable drinking water predominantly from communal outdoor taps, however; untreated borehole water and water from a river or stream was also reported to be used. The majority of women from the Atlantic Ocean sites used potable water from indoor taps (69.7%), while only 30.3% made use of communal outdoor taps. More participants in the Atlantic Ocean sites were exposed to passive smoking in their households, compared to those from the Indian Ocean sites (49.5% *versus* 32.2%). Significantly more women from the Atlantic Ocean sites perceived air quality to be inferior in their neighbourhood, and reported environmental pollution around their homes.

### Lead levels in blood and urine of the study cohorts

Table 2 shows the maternal PbB concentrations in the total study sample and by population residing along the Indian and the Atlantic Ocean coastal areas.

**Table 2:** Maternal blood lead (PbB) concentrations (μg/dL)—total and by Indian and Atlantic Ocean geographical position.

	Total (n = 640)	Indian Ocean (n = 350)	Atlantic Ocean (n = 290)	P value
**Maternal PbB levels (μg/dL)**				<0.001
**Mean**	1.77	2.21	1.23	
**SD**	1.85	2.23	1.02	
**Geometric mean**	1.32	1.73	0.95	
**95% CI**	1.24–1.40	1.60–1.86	0.88–1.03	
**Median**	1.40	1.72	1.0	
**Range**	0.04–31.7	0.04–31.7	0.5–6.2	
**IQR**	1.48	1.39	1.09	

In the total study sample, the geometric mean (GM) concentration of maternal PbB was 1.32μg/dL (n = 640; 95% CI, 1.24–1.40), with women residing along the Indian Ocean having significantly higher PbB levels, of GM 1.73 μg/dL (n = 350; 95% CI, 1.60–1.86) *versus* GM 0.95 μg/dL (n = 290; 95%CI, 0.88–1.03) of women residing along the Atlantic Ocean coast (Wilcoxon rank sum, p<0.001).

The concentrations and descriptive statistics for Pb levels in paired maternal and cord blood samples and respective pre-delivery maternal urine samples in the subset cohort residing along the Indian Ocean sites are shown in Table 3 (total for the Indian Ocean study population and by specific study site). Individual results can be accessed in supporting information S1 Individual data.

**Table 3:** Lead concentrations in paired maternal and cord blood samples, and maternal urine samples at delivery in the Indian Ocean subset cohort (total and by study site).

Indian Ocean Sites	Statistics	Maternal Blood Pb (μg/dL)	Cord Blood Pb (μg/dL)	Maternal Urine Pb(μg/L)	Maternal urine Creatinine-corrected (Pb μg/g creatinine)
**Total**	Mean	2.21	1.55	1.97	2.30
**N = 317**	SD	2.22	1.19	2.01	3.22
	Median	1.72	1.23	1.34	1.61
	GM	1.73	1.26	1.43	1.63
	95% Conf. Interval	1.60–1.86	1.18–1.35	1.31–1.56	1.49–1.78
	Min	0.04	0.14	0.10	0.18
	Max	31.70	10.24	15.9	49.19
	90^th^ percentile	3.92	2.72	4.10	4.32
**Site 1**	Mean	3.10	2.14	2.20	2.27
**N = 77**	SD	3.49	1.74	2.09	2.32
	Median	2.52	2.37	1.40	1.34
	GM	2.37	1.71	1.59	1.59
	95% Conf. Interval	2.07–2.71	1.48–1.98	1.33–1.90	1.33–1.90
	Min	0.33	0.57	0.22	0.26
	Max	31.70	10.24	12.34	12.0
	90^th^ percentile	5.12	4.04	4.95	4.79
**Site 2**	Mean	1.87	1.33	2.02	2.52
**N = 191**	SD	1.30	0.74	2.14	3.87
	Median	1.60	1.21	1.39	1.85
	GM	1.51	1.16	1.44	1.73
	95% Conf. Interval	1.37–1.67	1.07–1.25	1.28–1.62	1.53–1.95
	Min	0.04	0.14	0.10	0.18
	Max	10.73	4.64	15.9	49.19
	90^th^ percentile	3.18	2.29	4.06	4.56
**Site 3**	Mean	1.83	1.45	1.42	1.55
**N = 49**	SD	1.28	1.28	1.06	0.80
	Median	1.39	1.09	1.12	1.47
	GM	1.55	1.11	1.19	1.36
	95% Conf. Interval	1.33–1.81	0.90–1.38	1.01–1.40	1.17–1.59
	Min	0.68	0.14	0.36	0.41
	Max	6.40	7.90	6.38	4.69
	90^th^ percentile	4.09	3.24	2.65	2.53

The GM of maternal PbB in the total subset cohort was 1.73 μg/dL (n = 350; 95% CI, 1.60–1.86), with women residing at Site 1 having the highest GM PbB concentration of 2.37 μg/dL (n = 100; 95% CI, 2.07–2.71), followed by 1.55 μg/dL (n = 50; 95% CI, 1.33–1.81) and 1.51 μg/dL (n = 200; 95% CI, 1.37–1.67) in Site 3 and Site 2, respectively (Kruskal-Wallis rank test, p<0.001).

The GM level of PbC in the three Indian Ocean sites (total sample) was 1.26 μg/dL (n = 317; 95% CI, 1.18–1.35), see Table 3. A positive correlation was found between the log maternal PbB and the log cord blood Pb (PbC): Log (cord blood Pb) = 0.92 + 0.57 x Log (maternal blood Pb) rho = 0.65, p = <0.001).

The GM concentration of Pb in maternal urine (PbU) in the subset-cohort was 1.43 μg/L (n = 318; 95% CI, 1.31–1.56) and the creatinine-corrected GM for PbU levels was 1.63 μg/g creatinine (n = 318; 95% CI, 1.49–1.78). Women residing in Site 1 had the highest uncorrected creatinine PbU concentration of 1.59 μg/L.

Of the total cord blood samples, 4.4% (n = 14) had a BLL of >5μg/dL and only 1.3% (n = 4) had a BLL of >10μg/dL.

### Obstetric and neonate parameters

Table 4 shows obstetric and neonate parameters for the total sample and differences between the Indian and Atlantic Ocean coast study sites.

**Table 4:** Obstetric and neonate parameters.

Characteristic/Parameter	Total (n = 650)	Indian Ocean (n = 350)	Atlantic Ocean (n = 300)	p-value
**Maternal weight (kg)(GM, 95% CI^)**	71.83 (70.66–73.02)	72.14 (70.86–73.44)	71.47 (69.42–73.58)	0.501
**Maternal height (m)(GM, 95% CI^)**	1.57 (1.56–1.58)	1.57 (1.56–1.59)	1.58 (1.56–1.59)	0.126
**Gestational age (weeks)(GM, 95% CI^)**	38.16 (37.97- 38.36)	37.79 (37.60–37.99)	38.62 (38.27- 38.98)	<0.001*
**Birth weight (g) (GM, 95% CI^)**	3014.56 (2970.33–3059.46)	3009.51 (2952.66–3067.46)	3020.28 (2951.38–3090.79)	0.243
**Birth length (cm) (GM, 95% CI^)**	49.37 (49.08- 49.66)	49.10 (48.75–49.46)	49.67 (49.20–50.14)	0.019*
**Head circumference (cm) (GM, 95% CI^)**	34.66 (34.50–34.82)	35.00 (34.81–35.21)	34.27 (34.02–34.53)	<0.001*
**Apgar score 1min (mean, SD)**	8.8 (7.5)	8.6 (1.0)	9.0 (10.8)	0.038*
**Apgar score 5min (mean, SD)**	9.7 (0.8)	9.9 (0.7)	9.6 (0.8)	<0.001*
**Sex (%male)(GM, 95% CI^)**	52.8 (48.8–56.7)	50.9 (45.5–56.3)	54.9 (49.1–60.8)	0.320
**Parity (n, %, 95% CI^)**				
**0**	43.5 (39.7–47.4)	49.1 (43.8–54.5)	36.9(31.3–42.6)	
**1+**	56.5 (52.6–60.4)	50.9 (45.5–56.2)	63.1 (57.5–68.7)	0.002*

* Indicates significance (p<0.05)

^^^CI–Confidence Interval

Significant differences in the gestational age between Indian and Atlantic Ocean site populations were found, with neonates from the Indian Ocean sites having a shorter gestational age (p<0.001). Birth length and head circumference differed significantly between the Indian and Atlantic Ocean sites (p = 0.019 and p<0.001, respectively). Mothers at the Indian Ocean sites had significantly higher Apgar scores at 5 min (p<0.001). Forty nine percent of mothers from the Indian Ocean sites were primiparous, as compared to 36.9% at the Atlantic Ocean sites (p = 0.002). Eleven percent of infants in the total cohort were considered to be of low birth weight (<2500g). However, no significant differences were observed in birth weight between the sites. Maternal height and weight at delivery did not differ between sites. No significant differences were found in sex ratio of neonates between sites, with 50.9% and 54.9% being males at the Indian and Atlantic Ocean sites, respectively.

In the bivariate analysis of association between Pb exposures, maternal covariates and infant anthropometry measures at birth, it was found that maternal PbB was not correlated with the weight, length nor head circumference of the infants. However, the birth weight and length of infants were positively correlated with maternal age. In addition, weight of baby was positively correlated with parity, maternal weight and maternal height. Infant head circumference was also positively correlated with PbC levels, see Table 5.

**Table 5:** Spearman’s rank correlation coefficient (p-value) of associations between exposures, maternal covariates and infant anthropometry measures at birth.

Variable	Infant anthropometry measures			Maternal blood Pb (μg/dL)
	Weight (g)	Length (cm)	Head circumference (cm)	
**Maternal blood Pb**	0.058(0.413)	-0.106 (0.135)	0.083 (0.239)	-
**Urinary Pb (PbU)**	0.054 (0.450)	-0.139 (0.048)*	0.103 (0.145)	0.410 (<0.001)*
**Cord Pb (PbC)**	0.110 (0.120)	-0.056 (0.426)	0.149 (0.034)*	0.742 (<0.001)*
**Creatinine corrected Pb**	0.011 (0.880)	-0.151 (0.033)*	0.095 (0.178)	0.336 (<0.001)*
**Age**	0.227 (0.001)*	0.195 (0.006) *	0.084 (0.235)	-0.103 (0.146)
**Parity**	0.141 (0.045)*	0.089 (0.210)	0.076 (0.283)	-0.033 (0.646)
**Maternal weight**	0.171 (0.015)*	0.131 (0.063)	0.057 (0.423)	-0.190 (0.007)*
**Maternal height**	0.224 (0.001)*	0.074 (0.298)	0.081 (0.250)	0.135 (0.055)

* Indicates significance (p<0.05)

An association was also found between maternal PbB and PbC (rho = 0.742; p<0.001); as well as maternal PbB and maternal weight (rho = -0.190; p = 0.007); maternal PbB and maternal PbU, both for uncorrected (rho = 0.410; p<0.001) and creatinine-corrected concentrations of Pb (rho = 0.336; p<0.001).

In addition, to investigate whether any gender related differences existed in birth outcomes in response to maternal PbB levels, the investigators repeated the bivariate analysis for male (Table 6) and female (Table 7) neonates separately.

**Table 6:** Spearman’s rank correlation coefficient (p-value) of associations between exposures, maternal covariates and infant anthropometry measures at birth in male neonates and their mothers.

Variable	Male neonate anthropometry measures			Maternal blood Pb (μg/dL)
	Weight (g)	Length (cm)	Head circumference (cm)	
Maternal blood (PbB)	0.088 (0.374)	-0.073 (0.464)	0.012 (0.902)	-
Urinary Pb (PbU)	0.058 (0.559)	-0.094 (0.341)	0.100 (0.330)	0.387 (<0.001)*
Cord Pb (PbC)	0.124 (0.209)	-0.059 (0.552)	0.069 (0.486)	0.786 (<0.001)*
Creatinine corrected Pb	-0.052 (0.598)	-0.179 (0.069)	0.033 (0.744)	0.319 (0.001)*
Age	0.268 (0.006)*	0.231 (0.019)*	0.042 (0.676)	-0.222 (0.024)*
Parity	0.258 (0.008)*	0.169 (0.086)	0.050 (0.612)	-0.113 (0.255)
Maternal weight	0.247 (0.011)*	0.304 (0.002)*	0.147 (0.137)	-0.218 (0.026)*
Maternal height	0.268 (0.006)*	0.180 (0.068)	0.103 (0.299)	0.155 (0.117)

* Indicates significance (p<0.05)

**Table 7:** Spearman’s rank correlation coefficient (p-value) of associations between exposures, maternal covariates and infant anthropometry measures at birth in female neonates and their mothers.

Variable	Female neonate anthropometry measures			Maternal blood Pb (μg/dL)
	Weight (g)	Length (cm)	Head circumference (cm)	
Maternal blood Pb	0.003 (0.978)	-0.170 (0.095)	0.135 (0.185)	-
Urinary Pb (PbU)	0.081 (0.427)	-0.183 (0.072)	0.122 (0.230)	0.418 (<0.001)*
Cord Pb (PbC)	0.100 (0.330)	-0.054 (0.595)	0.243 (0.016)*	0.700 (<0.001)*
Creatinine corrected Pb	0.090 (0.379)	-0.134 (0.188)	0.151 (0.138)	0.348 (<0.001)*
Age	0.188 (0.064)	0.153 (0.132)	0.144 (0.158)	0.028 (0.787)
Parity	-0.006 (0.954)	-0.024 (0.816)	0.073 (0.478)	0.054 (0.597)
Maternal weight	0.101 (0.321)	-0.050 (0.623)	-0.005 (0.961)	-0.167 (0.101)
Maternal height	0.189 (0.063)	-0.037 (0.717)	0.095 (0.351)	0.126 (0.217)

* Indicates significance (p<0.05)

It was found that in male neonates, no correlation was evident between maternal PbB levels and infant weight, length nor head circumference. However, birth weight of male neonates was positively associated with maternal age (rho = 0.268; p = 0.006), weight (rho = 0.247; p = 0.011), as well as with parity (rho = 0.258; p = 0.008). The birth length was also positively associated with maternal age (rho = 0.231; p = 0.019) and weight (rho = 0.304; p = 0.002), but no associations were detected for head circumference (Table 6).

In female neonates, a positive association was found between PbC levels and head circumference (rho = 0.243; p = 0.016), but none for other factors (Table 7).

The bivariate analysis between maternal PbB levels and selected covariates that could contribute or be a source of Pb exposure are shown in Table 8.

**Table 8:** Means of maternal PbB levels by different characteristics of the study population along with crude β coefficients and p-values.

Characteristic	Number of Observation (N)	Maternal blood PbB μg/dL Geometric Mean (95% Conf. Interval)	Crude β coefficient (95% Conf. Interval)	p-value
**Race**				
African/Black	494	1.43 (1.33–1.53)	Reference	
Other	132	1.00 (0.89–1.13)	-0.35 (-0.50 to -0.21)	<0.001
**Education**				
None/Primary	54	2.13 (1.76–2.58)	Reference	
Secondary	396	1.16 (1.07–1.24)	-0.62 (-0.83 to -0.41)	<0.001
Tertiary	166	1.47 (1.30–1.66)	-0.37 (-0.60 to -0.15)	0.001
**Source of drinking water**				
Indoor tap	236	1.00 (0.92–1.10)	Reference	
Outdoor tap	328	1.46 (1.35–1.59)	0.38 (0.26 to 0.50)	<0.001
Borehole	32	2.29 (1.81–2.90)	0.82 (0.56 to 1.09)	<0.001
River/stream	29	1.92 (1.54–2.39)	0.65 (0.37 to 0.93)	<0.001
**Location of study site**				
Site 1	100	2.37 (2.07–2.71) 1	Reference	
Site 2	200	1.51 (1.37–1.67)	-0.45 (-0.62 to-0.28)	<0.001
Site 3	50	1.55 (1.33–1.81)	-0.42 (-0.66 to-0.19)	<0.001
Site 4	195	0.95 (0.86–1.05)	-0.91 (-1.08 to-0.75)	<0.001
Site 5	95	0.95 (0.84–1.08) 4	-0.92 (-1.11 to -0.72)	<0.001
**Residence**				
Urban/Informal settlement	80	0.99 (0.84–1.17)	Reference	
Rural	319	1.76 (1.63–1.90) 8	0.58 (0.40 to 0.75)	<0.001
Peri-urban	202	0.95 (0.86–1.05)	-0.04	0.678
**Cooking fuel**				
Electricity	471	1.16 (1.09–1.25)	Reference	
Paraffin	33	1.35 (1.08–1.69)	0.15(-0.11 to 0.40)	0.150
Gas/wood	127	2.10 (1.88–2.36) 1	0.59 (0.45 to 0.74)	<0.001
**Heating fuel**				
Electricity	284	1.21 (1.11–1.32)	Reference	
Paraffin	76	1.03 (0.88–1.20)	0-.16 (-0.35 to 0.03)	0.095
Gas/wood/coal	87	1.54 (1.31–1.80)	0.24 (0.06 to 0.42)	0.008
None	154	1.74 (1.54–1.97)	0. 37 (0.22 to 0.51)	<0.001
**Malaria control**				
Yes	106	2.33 (2.04–2.65)	Reference	
No	335	1.34 (1.24–1.44)	-0.55 (-0.71 to -0.40)	<0.001
**Pesticides use at home**				
Yes	445	1.39 (1.29–1.49)	Reference	
No	181	1.17 (1.0–1.30)	-0.17 (-0.30 to -0.04)	0.011
**Dairy products before pregnancy**				
Seldom	84	1.62 (1.36–1.92)	Reference	
At least once/week	105	1.40 (1.19–1.66)	-0.141 (-0.356 to 0.075)	0.201
Almost everyday	369	1.20 (1.12–1.29)	-0.297 (-0.475 to -0.120)	<0.001
**Dairy products during pregnancy**				
Seldom	86	1.58 (1.34–1.87)	Reference	
At least once/week	98	1.40 (1.17–1.68)	-0.122 (-0.340 to 0.095)	0.268
Almost everyday	367	1.19 (1.11–1.28)	-0.288 (-0.464 to -0.112)	0.001
**Butter and cheese before pregnancy**				
Seldom	147	1.61 (1.43–1.81)	Reference	
At least once/week	153	1.33 (1.18–1.51)	-0.188 (-0.352 to -0.023)	0.025
Almost everyday	175	1.10 (0.99–1.21)	-0.383 (-0.542 to -0.224)	<0.001
**Butter and cheese during pregnancy**				
Seldom	151	1.61 (1.43–1.81)	Reference	
At least once/week	148	1.32 (1.16–1.50)	-0.198 (-0.364 to -0.033)	0.019
Almost everyday	180	1.10 (0.99–1.21)	-0.385 (-0.544 to -0.227)	<0.001

* Indicates significance (p<0.05)

The levels of maternal PbB were higher among Black African mothers than in the other racial groups (Geometric mean 1.43 μg/dL *versus* 1.00 μg/dL; p<0.001) and highest in the mothers with the lowest educational levels (2.13 μg/dL). Mothers who sourced drinking water from communal taps, borehole or river had significantly higher PbB levels than those with indoor taps. Participants residing in study Site 1 had the highest PbB concentrations (mean 2.37 μg/dL: p<0.001), when compared to mothers residing in other sites. There was no significant difference in the levels of the maternal PbB with respect to the gender of the neonate (p = 0.808).

Maternal PbB concentrations were also highest among mothers who used either wood or gas as cooking fuel, instead of other sources of fuel, such as electricity and paraffin (Geometric mean 1.54 μg/dL: Crude β: 0.24; p = 0.008). Use of pesticides at home also contributed to higher PbB concentrations in mothers than not using pesticides at home (Geometric mean 1.39 μg/dL vs. 1.17 μg/dL: p = 0.011). There was no significant difference in the levels of maternal PbB between mothers who resided in households where somebody used paint at home, or not. No effect of smoking or exposure to second hand smoking or various types of dietary intake was detected.

Mothers who consumed dairy products such as milk before pregnancy or during pregnancy almost every day had lower levels of Pb in their blood than mothers who seldom had dairy products during any of the aforementioned periods.

In the final multivariate linear regression model (Table 9), mothers who reported they did not use pesticides at home had lower PbB levels, compared to mothers who reported using pesticides at home (β = -0.14; 95% CI, -0.26 to -0.02).

**Table 9:** Linear regression model of factors associated with maternal PbB (log transformed PbB).

Characteristic	Adjusted β	95% Conf. Interval	p-value
**Pesticide use at home**			
Yes	1.0		
No	-0.14	-0.26 to -0.021	0.022
**Location**			
Site 1	1.0		
Site 2	-0.37	-0.55 to -0.19	<0.001
Site 3	-0.35	-0.59 to -0.11	0.005
Site 4	-0.79	-0.98 to -0.61	<0.001
Site 5	-0.80	-1.01 to -0.60	<0.001
**Education**			
None/Primary	1.0		
Secondary	-0.28	-0.49 to -0.064	0.011
Tertiary	-0.25	-0.47 to -0.03	0.026

Mothers from study Sites 2, 3, 4 and 5 were more likely to have lower PbB levels compared to mothers residing in Site 1. Mothers with secondary and tertiary education (β = -0.28; 95% CI, -0.49 to -0.064 and β = -0.25; 95% CI, -0.47 to -0.03; respectively) were shown to have lower PbB levels, compared to mothers with low educational levels.

## Discussion

The current study has assessed exposure to Pb *in utero* in populations residing along the coastal regions of both the Indian and Atlantic Oceans in South Africa. The study has also examined the effects of prenatal exposure to Pb on birth outcomes, and identified contributing socio-economic and lifestyle factors. To the best of the authors’ knowledge, this is the first study to date investigating these associations in a large cohort of South African women after final removal of Pb from countries petrol in 2006.

Overall, highly significant differences in PbB levels were found between the study populations from the Indian and Atlantic Ocean sites (p<0.001), as well as between geographical sites. Women residing along the Atlantic Ocean had much lower PbB levels than Indian Ocean residents. Industrial and agricultural activities are more intense in the Indian Ocean region and different climatic conditions between the two regions may well be contributing factors.

Women residing in study Site 1 along the Indian Ocean had significantly higher levels of PbB, compared to women living in the other sites that formed part of this study. In the subset cohort of 317 women residing along the Indian Ocean, Pb was measured in three parameters, namely paired maternal (PbB) and cord (PbC) blood and maternal urine (PbU). The GM maternal PbB was 1.73μg/dL (mean 2.21 (SD 2.22) μg/dL and the cord blood GM was 1.26 μg/dL (mean 1.55 (SD 1.19) μg/dL). In the earlier South African study performed by Karimi et al. in 1996 in two Durban hospitals, also situated along the Indian Ocean, the mean (SD) concentrations of Pb in paired maternal and cord blood samples were 7.35 (3.85) μg/dL and 6.56 (3.1) μg/dL, respectively. Of these, 18.7% and 11.3% of paired maternal and cord blood samples, respectively, exceeded the CDC cut off value for Pb of 10 μg/dL [18]. When comparing these results with the results from the current study (both studies having been carried out in the same geographical region, although many years apart), a significant reduction was found in *in utero* exposure to Pb, along the Indian Ocean sites. Unfortunately, there are no earlier prenatal studies from the Atlantic sites which can be compared to the results in the current study.

A linear positive correlation was found between log maternal and cord blood Pb levels confirming the ability of Pb to cross the placental barrier (rho = 0.65, p = <0.001). This finding is in agreement with other published studies [31, 32].

Consensus has not been reached regarding the correlation between Pb levels in blood and urine. The current study has shown a low but statistically significant correlation with Pb levels in blood and urine samples. Some studies have shown a correlation of Pb levels in blood and urine for occupational but not for environmental exposure; while other studies have confirmed the correlation of Pb levels to occupational exposure, but not close enough to allow for PbU concentrations to predict PbB levels on an individual basis [33,34].

The maternal PbU concentrations in the current study are far lower than those reported from an industrial area in Taranto, Italy (median 7.3 μg/L); the general population in Thailand (GM 2.54 μg/g creatinine); women from the general population in Japan (GM 2.18 μg/g creatinine); and from children in Kinshasa (GM 2.88 μg/g creatinine) [35–38].

The present study has found the maternal PbB concentrations to be considerably lower when compared to other developing countries. For example, Pb levels in paired maternal and cord blood samples in Shanghai (China) were reported to be 4.34 μg/dL and 2.64 μg/dL, respectively; in Mumbai (India) 6.4 μg/dL and 5.1μg/dL, respectively; and in Mexico City (Mexico) 5.7 μg/dL and 6.8 μg/dL, respectively [39–41]. There is a paucity of data from other African countries but indications are that prenatal exposure to Pb is fairly common. A recent study from Eastern Nigeria has reported paired maternal and cord blood Pb levels of 6.19 μg/dL and 4.75 μg/dL, respectively [42].

A number of published studies from developed countries indicate a steady lowering of PbB levels in both general and susceptible populations. Studies from the USA (New York) have found maternal and cord blood Pb levels of 1.96 μg/dL and 1.65 μg/dL, respectively; in Canada (Montreal) 2.1 μg/dL and 1.7 μg/dL, respectively; in Sweden 1.14 μg/dL and 1.12 μg/dL, respectively; and in Taiwan 1.58 μg/dL and 1.29 μg/dL, respectively [5, 43–45]. These Pb concentrations are very similar to the low values found in the South African populations in our study.

Historically, leaded petrol in SA contained high concentration of lead (0.836 g/l) and its removal took place in stages over many years. The unique socio-ecological features of the SA population suggest that the trajectory of change in PbB will likely differ from those reported in Europe or North America [24].

On investigation of the association between Pb exposure, maternal covariates and infant anthropometry, no effects on birth weight, length and head circumference were observed. Other epidemiological studies have reported significant associations between maternal PbB levels and birth weight, at low level Pb exposure [46,47]. Nonetheless, in the present study, the birth weight and length of infants were positively correlated with maternal age, and weight of infants was positively correlated with parity, maternal weight and maternal height. Infant head circumference was also positively correlated with PbC levels and parity.

This study also evaluated gender birth outcomes in response to maternal PbB, but no correlation was evident in male neonates. On the other hand, in female neonates, a positive association was identified between PbC levels and head circumference. This finding is in agreement with Tang et al.2016, but differed from Wang et al. 2017, who found a significant inverse association between PbC levels and head circumference, in the male neonate subgroup only [48,49].

The present study has also examined possible socio-economic and behavioural/lifestyle factors that may have influenced exposure to Pb. As expected, race, socio-economic and educational status of mothers had significant impact on PbB levels in this study population. Levels of maternal PbB were found to be higher among Black African mothers than in the other racial groups, and highest in mothers with a low educational level. In addition, mothers who sourced drinking water from communal taps, borehole or river had significantly higher PbB levels. Maternal PbB levels were also highest among mothers who used either wood or gas as cooking fuel. Use of pesticides at home also contributed to higher PbB concentrations in mothers. There was no significant difference in the levels of maternal PbB between mothers who resided in households where somebody used paint at home or not. No effects of smoking or exposure to second hand smoking or various types of dietary intake were detected. Alcohol consumption by a low number of participants did not show any association either.

An in-depth evaluation of the findings of the current study has shown low concentrations of Pb in biological fluids of delivering women, indicating low *in utero* exposure to Pb, when compared to earlier studies in South Africa and elsewhere. The final removal of Pb from South African fuels in 2006 and the introduction of lead-free paint in 2009 may well be the main explanatory factor. Our studies were performed in 2008 along the Indian Ocean coast and in 2012–2013 along the Atlantic Ocean coast; both timeframes were a few years after the final removal of Pb from fuels, in line with new legislation.

There are number of limitations in the study that need to be considered. The first is a recall bias in dietary intake. Secondly, due to financial constraints, the collection of cord blood samples was limited to women residing along Indian Ocean coast only, which may have introduced some bias.

## Conclusions

The present study has characterised the *in utero* exposure to Pb in a large cohort of South African women residing along coastal regions. This investigation has demonstrated not only the positive impact that the introduction of unleaded petrol and lead-free paint has had on *in utero* exposure to Pb in South Africa, but has also contributed new data on the topic, in a region where such data and scientific investigations in this field are lacking. Future research should evaluate if similar effects can be detected in young children and the adult population. The findings of this study are invaluable to policy development and will be communicated to the national departments of environmental affairs and public health.

The well documented scientific evidence for the toxic and irreversible health effects of Pb even at very low levels as found in our study, have over the past few decades resulted in action from governments worldwide. As such, many developed nations became frontrunners in banning Pb in petrol, resulting in sharp and steady declines in blood Pb levels in their general populations.

It is imperative that known sources of lead exposure must be removed in developing countries as continued exposures will certainly lead to detrimental health effects, the impacts of which may well take many years to manifest, and can be expected to be particularly harmful in vulnerable populations, such as pregnant women and their developing foetuses.

## Supporting information

S1 Individual data (DOCX)
