# Supplementary material for: Reduction of in utero lead exposures in South African populations: Positive impact of unleaded petrol
Source: PLoS One. 2017 Oct 16;12(10):e0186445. doi: 10.1371/journal.pone.0186445 (PMC5643117; doi:10.1371/journal.pone.0186445)
Supplement: S1 Individual data — (DOCX) [file pone.0186445.s001.docx]

**Individual results for maternal Pb (mwpb ug/L); cord blood Pb (cpb ug/L); maternal urine Pb (pbu ug/L); maternal urine Pb creatinine corrected (pbcreat ug/g creatinine)**

| mwpb | cpb | pbu | pbcreat |  |
| --- | --- | --- | --- | --- |
| 10.15 | 7 | 0.872 | 1.32 |  |
| 27.9 |  | 1.556 | 1.2 |  |
| 18.01 |  | 0.853 | 1.05 |  |
| 11.98 | 6.9 | 0.316 | 0.39 |  |
| 37.15 | 20.9 | 4.14 | 1.79 |  |
| 88.74 | 59.1 | 5.787 | 9.04 |  |
| 29.1 | 22.3 | 3.812 | 2.48 |  |
| 26.66 | 17.6 | 4.957 | 4.24 |  |
| 27.72 | 14 | 7.289 | 9.47 |  |
| 43.43 |  |  |  |  |
| 29.25 | 24.5 | 2.461 | 1.94 |  |
| 19.24 |  |  |  |  |
| 39.28 | 34.7 | 0.725 | 0.725 |  |
| 17.15 | 12.9 | 0.88 | 0.76 |  |
| 51.24 | 27.4 |  |  |  |
| 14.59 |  | 0.725 | 1.34 |  |
| 8.252 | 8 | 1.805 | 0.83 |  |
| 9.841 |  | 1.055 | 0.58 |  |
| 14.85 |  | 1.006 | 1.18 |  |
| 32.85 | 24.2 | 3.079 | 1.37 |  |
| 12.09 | 7.3 | 1.215 | 0.69 |  |
| 120.4 | 102.4 | 1.883 | 0.87 |  |
| 16.48 | 8.2 | 0.865 | 0.48 |  |
| 3.249 | 5.7 | 0.694 | 1.18 |  |
| 18.75 | 17.2 | 2.396 | 3.74 |  |
| 23.69 | 23.9 | 2.357 | 1.64 |  |
| 9.913 | 9.3 | 0.216 | 0.72 |  |
| 32.11 | 19.6 |  |  |  |
| 11.5 | 10.6 | 0.964 | 0.58 |  |
| 15.61 | 9.9 | 1.27 | 1.2 |  |
| 29.84 |  | 0.642 | 0.642 |  |
| 27.58 |  |  |  |  |
| 11.09 | 11.4 |  |  |  |
| 9.387 | 10.2 | 0.972 | 0.63 |  |
| 8.838 | 9.4 | 1.974 | 1.69 |  |
| 6.205 | 6.3 |  |  |  |
| 14.6 |  | 0.909 | 0.909 |  |
| 29.68 | 15.9 | 2.347 | 2.04 |  |
| 27.36 | 13.9 | 4.817 | 3.95 |  |
| 16.64 | 12.2 | 1.253 | 1.17 |  |
| 38 |  | 4.857 | 2.76 |  |
| 23.73 | 17.9 | 1.529 | 2.04 |  |
| 46.29 | 50.4 | 5.525 | 3.41 |  |
| 32.7 | 25.9 | 5.398 | 12 |  |
| 31.57 |  | 5.096 | 3.61 |  |
| 17.11 | 10.3 | 1.346 | 1.346 |  |
| 30.86 | 10 | 0.388 | 0.26 |  |
| 25.67 |  |  |  |  |
| 12.82 | 7.3 | 2.007 | 2.007 |  |
| 21.69 | 15.1 |  |  |  |
| 18.91 | 15.5 | 2.497 | 5.31 |  |
| 33.47 |  | 1.319 | 1.4 |  |
| 68.98 | 49.7 | 3.215 | 8.46 |  |
| 65.09 |  | 1.309 | 2.62 |  |
| 43.08 | 55.6 | 1.633 | 4.8 |  |
| 36.2 | 15.5 | 1.377 | 1.377 |  |
| 13.12 |  | 2.068 | 2.43 |  |
| 11.98 |  | 1.275 | 1.52 |  |
| 24.11 | 11.6 |  |  |  |
| 33.88 | 28.5 | 2.573 | 9.9 |  |
| 14.47 | 11.6 | 1.919 | 1.24 |  |
| 15.17 |  | 2.178 | 1.04 |  |
| 16.5 | 32.9 |  |  |  |
| 10.69 | 10 | 1.002 | 0.64 |  |
| 19.89 | 16.2 | 2.816 | 1.2 |  |
| 30.9 | 19.3 | 1.965 | 2.85 |  |
| 14.85 | 10.7 | 1.386 | 0.89 |  |
| 40.38 | 27.2 | 1.244 | 1.244 |  |
| 10.35 | 8 | 1.311 | 1.21 |  |
| 8.4 |  | 0.236 | 0.49 |  |
| 16.74 | 7.7 |  |  |  |
| 41.02 | 37.8 |  |  |  |
| 17.28 | 11.5 | 0.404 | 0.26 |  |
| 19.32 | 13.3 | 0.802 | 1.31 |  |
| 34.31 | 13 | 1.292 | 1.292 |  |
| 53.51 | 42.3 | 10.2 | 4.74 |  |
| 50.71 | 30.1 |  |  |  |
| 29.11 | 23.5 | 4.386 | 4.57 |  |
| 32.13 | 23.2 | 1.805 | 2.03 |  |
| 11.89 |  | 2.471 | 1.28 |  |
| 28.5 | 23.2 | 2.315 | 2.34 |  |
| 18.37 | 9.9 | 0.919 | 0.919 |  |
| 53.23 | 39.1 |  |  |  |
| 33.78 | 30 |  |  |  |
| 54.66 |  |  |  |  |
| 40.88 |  | 12.34 | 5.58 |  |
| 27.07 | 19.3 | 0.823 | 0.823 |  |
| 24.65 | 19.3 | 1.119 | 2.87 |  |
| 15.5 | 11.3 |  |  |  |
| 50.67 | 39.9 | 0.692 | 0.692 |  |
| 10.99 | 9.2 | 0.742 | 1.28 |  |
| 26.3 | 16.4 | 0.673 | 2.04 |  |
| 38.15 | 21.7 | 1.19 | 1.19 |  |
| 27.95 | 23.8 | 4.002 | 2.6 |  |
| 12.39 | 7.3 |  |  |  |
| 18.86 | 14.5 | 1.415 | 1.39 |  |
| 39.62 | 26.8 | 2.196 | 3.99 |  |
| 316.9 | 17.7 | 2.331 | 1.12 |  |
| 23.42 |  | 0.581 | 1.71 |  |
| 113.4 | 94.9 |  |  |  |
| 45.98 | 39.2 | 2.429 | 2.76 |  |
| 6.131 | 6.2 | 0.728 | 0.46 |  |
| 16.87 | 12.6 | 1.396 | 1.45 |  |
| 7.421 | 14.8 | 1.595 | 1.56 |  |
| 10.91 | 10.1 | 1.294 | 1.294 |  |
| 7.681 | 11.9 | 1.391 | 3.09 |  |
| 7.208 | 10.3 | 2.199 | 2.18 |  |
| 13.84 | 18.1 | 0.968 | 1.38 |  |
| 42.97 | 12.4 | 15.9 | 8.46 |  |
| 0.96 | 6.6 | 0.446 | 0.8 |  |
| 17.67 | 12.4 | 0.282 | 0.46 |  |
| 7.083 | 10.2 | 0.25 | 0.26 |  |
| 13.74 | 16.6 | 1.047 | 1.047 |  |
| 20.3 | 24.5 | 6.387 | 10.65 |  |
| 5.799 | 9.5 | 0.691 | 1.26 |  |
| 0.61 | 5.3 | 3.332 | 2.11 |  |
| 2.754 | 7.6 | 1.787 | 1.6 |  |
| 8.922 | 11.3 | 1.493 | 2.13 |  |
| 21.02 | 21.3 | 3.693 | 4.99 |  |
| 1.306 | 5.4 | 1.046 | 0.98 |  |
| 32.23 | 32.6 | 4.016 | 4.27 |  |
| 13.56 | 14.4 | 0.1 | 0.26 |  |
| 14.34 | 18.5 | 1.07 | 1.07 |  |
| 55.82 |  | 14.18 | 6.99 |  |
| 9.693 | 10.2 | 0.335 | 0.74 |  |
| 6.929 | 9.1 |  |  |  |
| 15.45 | 12.9 | 0.274 | 0.84 |  |
| 19.39 | 16.6 | 1.386 | 1.386 |  |
| 8.03 | 9.4 | 0.199 | 0.26 |  |
| 9.444 | 10.8 | 0.383 | 0.26 |  |
| 107.3 | 46.4 | 7.161 | 7.62 |  |
| 5.354 | 9.7 | 0.141 | 0.54 |  |
| 12.98 | 26.7 | 0.55 | 0.85 |  |
| 2.612 |  | 2.107 | 1.86 |  |
| 6.019 | 11.5 | 0.374 | 0.26 |  |
| 21.9 | 26.5 | 3.528 | 10.08 |  |
| 10.25 | 9.3 | 4.265 | 2.15 |  |
| 17.52 | 20.6 | 3.551 | 5.82 |  |
| 5.18 | 12 | 0.531 | 0.77 |  |
| 14.85 | 17.8 | 0.455 | 0.26 |  |
| 17.91 | 20.2 | 1.316 | 1.83 |  |
| 11.68 | 16.3 | 2.41 | 1.33 |  |
| 6.738 | 13.4 | 1.445 | 1.39 |  |
| 13.32 | 13.2 | 3.605 | 2.17 |  |
| 0.4 | 5.9 | 0.934 | 1.25 |  |
| 13.69 | 18.8 | 7.135 | 4.43 |  |
| 3.441 | 12.3 | 1.987 | 2.42 |  |
| 6.222 | 7.8 | 2.35 | 1.77 |  |
| 5.461 | 11.1 | 1.075 | 2.34 |  |
| 4.293 | 11.3 | 0.797 | 1.17 |  |
| 28.88 | 18.6 | 5.164 | 2.61 |  |
| 18.64 | 9.3 | 1.047 | 1.98 |  |
| 11.29 | 2.2 | 0.944 | 2.05 |  |
| 14.38 | 6.9 | 1.138 | 1.138 |  |
| 40.53 | 15.6 | 4.871 | 9.94 |  |
| 21.22 | 14.4 | 0.609 | 0.609 |  |
| 64.94 | 41.5 | 3.341 | 3.341 |  |
| 16.06 | 13.2 | 1.329 | 1.46 |  |
| 16.02 | 8.4 | 0.584 | 1.88 |  |
| 12.72 | 10.3 | 1.978 | 2.38 |  |
| 18.47 | 10.4 | 0.606 | 0.606 |  |
| 12.06 | 8.3 | 0.589 | 0.589 |  |
| 17.31 | 6 | 3.164 | 2.11 |  |
| 10.71 | 5 |  |  |  |
| 15.24 |  |  |  |  |
| 9.957 | 1.5 | 0.996 | 1.78 |  |
| 14.64 | 5.7 | 2.261 | 5.65 |  |
| 23.23 | 13.8 | 3.564 | 2 |  |
| 8.215 | 1.4 | 0.955 | 2.08 |  |
| 16.71 | 12.3 | 0.773 | 1.61 |  |
| 13.63 | 3.2 | 1.586 | 1.96 |  |
| 10.76 | 11.7 | 1.171 | 0.81 |  |
| 34.19 | 26.2 | 2.029 | 5.07 |  |
| 9.693 |  | 3.769 | 3.769 |  |
| 11 | 10.3 | 0.677 | 0.677 |  |
| 11.48 | 10.1 |  |  |  |
| 12.79 | 10.2 | 0.994 | 2.49 |  |
| 8.464 | 6.6 | 1.743 | 2.39 |  |
| 6.519 | 7.1 | 1.295 | 0.83 |  |
| 21.37 | 9.9 | 1.237 | 2.75 |  |
| 14.18 | 13.4 | 1.86 | 0.99 |  |
| 10.61 |  | 1.08 | 0.39 |  |
| 24.95 | 14.3 | 0.934 | 2.34 |  |
| 18.2 | 17.9 | 0.363 | 0.26 |  |
| 19.65 | 16.2 | 1.648 | 1.55 |  |
| 15.72 | 9.2 | 1.075 | 2.34 |  |
| 10.2 | 7.9 | 0.801 | 0.97 |  |
| 23.81 | 25.5 | 1.097 | 1.097 |  |
| 21.74 | 16.9 | 2.999 | 2.54 |  |
| 26.76 | 18.4 | 3.385 | 2.26 |  |
| 32.88 | 25.3 | 0.953 | 0.953 |  |
| 21.03 | 18.6 | 4.207 | 2.38 |  |
| 8.472 | 7 | 1.638 | 2.44 |  |
| 32.25 | 29.3 | 1.309 | 2.05 |  |
| 23.77 | 17.7 | 4.219 | 4.96 |  |
| 22.51 | 13.7 | 1.138 | 3.16 |  |
| 8.21 | 4.3 | 2.508 | 3.53 |  |
| 8.473 | 8.1 | 1.226 | 0.93 |  |
| 75.37 |  | 3.957 | 3.17 |  |
| 12.56 | 9.8 | 1.298 | 1.43 |  |
| 12.26 | 11.8 | 2.504 | 5.56 |  |
| 9.426 | 8.7 | 2.981 | 2.84 |  |
| 15.49 | 12 | 2.74 | 1.36 |  |
| 20.48 | 11.6 | 0.957 | 0.957 |  |
| 15.78 | 9.5 | 1.166 | 1.166 |  |
| 18.51 | 17.7 | 3.239 | 1.23 |  |
| 27.47 | 23.8 | 2.206 | 1.74 |  |
| 11.61 | 7.7 | 1.115 | 3.48 |  |
| 25.79 | 16.5 | 1.022 | 2.56 |  |
| 15.58 | 37.4 | 1.022 | 2.92 |  |
| 18.42 | 17.6 | 4.408 | 3.24 |  |
| 13.07 | 13.8 | 1.646 | 3.43 |  |
| 13.4 | 8.3 | 1.581 | 2.05 |  |
| 14.94 | 12.3 | 0.926 | 0.926 |  |
| 38.36 | 14.3 | 2.034 | 4.52 |  |
| 17.93 | 14.2 | 1.426 | 2.85 |  |
| 20.35 | 15.5 | 1.877 | 4.27 |  |
| 13.03 | 13.6 | 3.597 | 5.9 |  |
| 19.05 | 14.1 | 0.538 | 0.538 |  |
| 64.21 | 40.7 | 0.41 | 0.26 |  |
| 23.2 | 13 | 4.138 | 4.31 |  |
| 17.16 | 13.1 | 1.004 | 1.004 |  |
| 23.16 | 13.2 | 4.136 | 5.17 |  |
| 29.01 | 12.7 | 1.697 | 1.28 |  |
| 15.7 | 11.2 | 1.341 | 3.05 |  |
| 33.55 | 17.1 | 1.543 | 2.81 |  |
| 17.26 |  | 1.8 | 1.58 |  |
| 12.79 | 10.1 | 0.856 | 1.5 |  |
| 16.3 | 28.3 | 1.996 | 3.84 |  |
| 25.73 | 13 |  |  |  |
| 16.93 | 8.9 | 1.3 | 3.33 |  |
| 18.17 | 12 | 1.195 | 1.66 |  |
| 23.03 | 12 | 1.598 | 3.07 |  |
| 18.96 | 10.1 | 3.662 | 2.71 |  |
| 8.44 | 11.3 |  |  |  |
| 18.09 | 13.7 | 3.044 | 2.03 |  |
| 16.41 | 9.7 | 1.085 | 1.7 |  |
| 10.75 | 12.8 | 1.064 | 1.87 |  |
| 27.99 | 15.2 | 0.865 | 1.31 |  |
| 30.88 | 12.1 | 4.176 | 4.176 |  |
| 19.48 | 2.6 | 1.605 | 1.605 |  |
| 15.65 | 13.8 | 3.83 | 5.32 |  |
| 11.58 | 6.5 | 2.204 | 2.83 |  |
| 17.3 | 5.2 | 2.252 | 1.44 |  |
| 9.339 | 4.9 | 0.568 | 1.78 |  |
| 21.08 | 6.7 | 1.427 | 1.6 |  |
| 11.73 | 6.2 | 0.574 | 1.47 |  |
| 36.41 | 16.3 | 4.051 | 4.01 |  |
| 56.56 | 30.3 | 2.6 | 3.94 |  |
| 15.11 | 5.8 | 1.074 | 2.56 |  |
| 12.31 | 8.1 | 1.425 | 1.11 |  |
| 31.6 | 17.3 | 4.091 | 2.06 |  |
| 33 | 17.2 | 1.923 | 5.34 |  |
| 12.14 | 7.2 | 1.523 | 1.14 |  |
| 15.69 | 9.4 | 1.008 | 1.008 |  |
| 22.27 | 15.9 | 1.12 | 1.35 |  |
| 20 | 11.3 | 0.514 | 1.01 |  |
| 16.19 | 8 | 0.405 | 0.26 |  |
| 17.42 | 12.3 | 2.406 | 2.27 |  |
| 12.49 | 9.1 | 0.922 | 2 |  |
| 14.45 | 9.7 | 1.408 | 0.74 |  |
| 20.04 | 13.4 |  |  |  |
| 15.23 | 9.7 | 4.154 | 3.52 |  |
| 26.16 | 12.1 | 0.8 | 2.5 |  |
| 25.65 | 10.8 | 1.237 | 1.96 |  |
| 24.42 | 17.5 | 4.625 | 2.77 |  |
| 12.29 | 7.8 |  |  |  |
| 16.2 | 7.3 | 1.662 | 3.32 |  |
| 8.213 | 8.1 | 0.652 | 1.81 |  |
| 24.6 | 25 | 2.834 | 1.54 |  |
| 11.24 | 5.8 |  |  |  |
| 14.76 | 10.7 | 1.75 | 1.94 |  |
| 9.981 | 3.5 | 0.674 | 0.94 |  |
| 10.7 | 5.2 | 0.443 | 0.26 |  |
| 9.341 | 3.9 | 0.991 | 1.71 |  |
| 24.19 | 17 | 1.683 | 5.43 |  |
| 24.53 | 23.3 | 2.193 | 3.78 |  |
| 12.9 |  | 1.497 | 0.95 |  |
| 15.75 | 8.1 | 0.876 | 1.79 |  |
| 31.84 |  | 15.25 | 49.19 |  |
| 57.79 | 17.4 | 3.107 | 2.99 |  |
| 21.4 | 10.7 | 2.491 | 2.4 |  |
| 25.93 | 17.8 | 0.808 | 0.808 |  |
| 23.81 | 4.1 |  |  |  |
| 16.53 | 12.1 | 0.978 | 1.25 |  |
| 23.3 | 24.3 |  |  |  |
| 9.764 | 6.4 | 0.397 | 0.26 |  |
| 9.029 | 6.2 | 0.693 | 0.693 |  |
| 22.83 | 20.4 | 3.699 | 2.7 |  |
| 22.9 | 15.2 | 0.986 | 2.35 |  |
| 31.67 | 15.3 | 0.997 | 0.997 |  |
| 33.47 | 17.5 |  |  |  |
| 19.06 | 15.5 | 1.53 | 1.51 |  |
| 30.25 | 17.3 | 1.864 | 1.32 |  |
| 14.02 | 9 | 1.017 | 1.39 |  |
| 28.73 | 13.2 | 0.413 | 0.18 |  |
| 23.17 | 12.9 | 1.231 | 1.99 |  |
| 19.58 | 13.1 | 2.078 | 3.52 |  |
| 18.66 | 7 | 0.615 | 1.03 |  |
| 24.42 | 17.1 | 3.221 | 1.39 |  |
| 25.68 | 16.2 | 1.266 | 3.42 |  |
| 13.97 | 10.5 | 1.428 | 2.6 |  |
| 19.48 | 12.3 | 1.389 | 1.389 |  |
| 64.01 | 33.8 | 6.377 | 4.69 |  |
| 15.05 | 9.2 | 1.101 | 1.28 |  |
| 53.18 | 31.3 | 2.562 | 1.87 |  |
| 25.2 | 13.9 | 0.362 | 0.43 |  |
| 15.84 | 14.6 | 1.824 | 2.15 |  |
| 12.2 | 9.3 | 0.793 | 0.41 |  |
| 19.1 | 15.4 | 1.1 | 1.62 |  |
| 12 |  | 0.556 | 0.556 |  |
| 8.4 | 5.7 | 0.729 | 1.12 |  |
| 8.2 | 4.9 | 1.097 | 1.52 |  |
| 21.93 | 18.6 | 1.544 | 2.38 |  |
| 15.71 | 7.8 | 0.546 | 0.546 |  |
| 20.02 | 16.7 | 2.516 | 2.47 |  |
| 17.72 | 11 | 0.826 | 0.826 |  |
| 10.56 | 7.2 | 1.141 | 1.61 |  |
| 11.99 | 6.1 | 0.648 | 1.8 |  |
| 49.85 | 34.3 | 2.661 | 1.42 |  |
| 24.28 | 21.3 | 1.543 | 1.543 |  |
| 16.07 | 22.2 | 1.017 | 2.26 |  |
| 13.68 | 11.7 | 1.364 | 1.15 |  |
| 9.12 | 6.3 | 0.62 | 0.67 |  |
| 7.556 | 4 | 0.8 | 1.18 |  |
| 10.24 | 7.8 | 1.006 | 0.59 |  |
| 12.31 | 5.7 | 1.296 | 1 |  |
| 24.2 | 14.7 | 0.597 | 1.57 |  |
| 9.16 | 3.5 | 1.528 | 2.46 |  |
| 10.71 | 10.4 | 1.343 | 1.33 |  |
| 19.01 | 21 | 4.2 | 1.89 |  |
| 7.9 | 7 | 1.218 | 1.218 |  |
| 19.33 | 17 | 2.015 | 1.07 |  |
| 10.56 | 9.4 | 0.708 | 1.73 |  |
| 21.4 | 12.1 | 1.64 | 1.06 |  |
| 12.09 | 7.6 | 0.516 | 0.516 |  |
| 6.879 | 2.4 | 0.867 | 0.867 |  |
| 10.82 | 7.2 | 0.684 | 1.52 |  |
| 11.13 | 10.9 | 1.108 | 1.27 |  |
| 22.88 | 20.9 | 0.941 | 1.25 |  |
| 42.62 | 32.4 | 1.828 | 1.85 |  |
| 6.81 | 79 | 0.919 | 0.99 |  |
| 16.7 | 16.1 | 3.221 | 1.81 |  |
| 13.75 | 1.4 | 0.602 | 1.02 |  |
| 12.21 | 6.9 | 0.732 | 2.15 |  |
| 20.69 | 15 | 1.127 | 1.66 |  |
| 8.75 | 5.7 | 1.51 | 0.92 |  |
| 10 | 8.8 | 0.973 | 2.56 |  |
| 52.82 | 36.3 | 3.282 | 2.54 |  |
| 13.29 | 9.1 | 1.477 | 1.87 |  |
|  |  |  |  |  |
| 31 |  |  |  |  |
| 28 |  |  |  |  |
| 10 |  |  |  |  |
| 5 |  |  |  |  |
| 11 |  |  |  |  |
| 24 |  |  |  |  |
| 39 |  |  |  |  |
| 12 |  |  |  |  |
| 5 |  |  |  |  |
| 19 |  |  |  |  |
|  |  |  |  |  |
| 16 |  |  |  |  |
| 14 |  |  |  |  |
| 5 |  |  |  |  |
| 5 |  |  |  |  |
| 5 |  |  |  |  |
| 29 |  |  |  |  |
| 14 |  |  |  |  |
| 10 |  |  |  |  |
| 16 |  |  |  |  |
| 5 |  |  |  |  |
| 5 |  |  |  |  |
| 16 |  |  |  |  |
| 5 |  |  |  |  |
| 11 |  |  |  |  |
| 5 |  |  |  |  |
| 21 |  |  |  |  |
|  |  |  |  |  |
| 14 |  |  |  |  |
| 13 |  |  |  |  |
| 42 |  |  |  |  |
| 11 |  |  |  |  |
| 45 |  |  |  |  |
| 17 |  |  |  |  |
| 11 |  |  |  |  |
| 27 |  |  |  |  |
| 12 |  |  |  |  |
| 22 |  |  |  |  |
| 25 |  |  |  |  |
| 15 |  |  |  |  |
| 19 |  |  |  |  |
| 11 |  |  |  |  |
| 10 |  |  |  |  |
| 25 |  |  |  |  |
| 12 |  |  |  |  |
| 5 |  |  |  |  |
| 14 |  |  |  |  |
| 5 |  |  |  |  |
| 14 |  |  |  |  |
| 5 |  |  |  |  |
| 11 |  |  |  |  |
| 10 |  |  |  |  |
| 5 |  |  |  |  |
| 5 |  |  |  |  |
| 22 |  |  |  |  |
| 5 |  |  |  |  |
| 5 |  |  |  |  |
| 5 |  |  |  |  |
| 5 |  |  |  |  |
| 5 |  |  |  |  |
| 12 |  |  |  |  |
| 14 |  |  |  |  |
| 5 |  |  |  |  |
| 5 |  |  |  |  |
| 5 |  |  |  |  |
| 5 |  |  |  |  |
| 5 |  |  |  |  |
| 15 |  |  |  |  |
|  |  |  |  |  |
| 5 |  |  |  |  |
| 39 |  |  |  |  |
| 16 |  |  |  |  |
|  |  |  |  |  |
| 5 |  |  |  |  |
| 5 |  |  |  |  |
| 5 |  |  |  |  |
| 47 |  |  |  |  |
| 5 |  |  |  |  |
| 5 |  |  |  |  |
| 5 |  |  |  |  |
| 16 |  |  |  |  |
| 21 |  |  |  |  |
| 21 |  |  |  |  |
| 12 |  |  |  |  |
| 14 |  |  |  |  |
| 11 |  |  |  |  |
| 16 |  |  |  |  |
| 10 |  |  |  |  |
| 20 |  |  |  |  |
| 5 |  |  |  |  |
| 12 |  |  |  |  |
| 5 |  |  |  |  |
| 14 |  |  |  |  |
| 14 |  |  |  |  |
| 16 |  |  |  |  |
| 5 |  |  |  |  |
| 16 |  |  |  |  |
| 5 |  |  |  |  |
| 36 |  |  |  |  |
| 55 |  |  |  |  |
| 36 |  |  |  |  |
| 23 |  |  |  |  |
| 15 |  |  |  |  |
| 39 |  |  |  |  |
| 19 |  |  |  |  |
| 5 |  |  |  |  |
| 21 |  |  |  |  |
| 5 |  |  |  |  |
| 15 |  |  |  |  |
| 27 |  |  |  |  |
| 15 |  |  |  |  |
| 36 |  |  |  |  |
| 11 |  |  |  |  |
| 19 |  |  |  |  |
| 11 |  |  |  |  |
| 11 |  |  |  |  |
| 10 |  |  |  |  |
| 17 |  |  |  |  |
| 59 |  |  |  |  |
| 5 |  |  |  |  |
| 19 |  |  |  |  |
| 10 |  |  |  |  |
| 13 |  |  |  |  |
| 12 |  |  |  |  |
| 24 |  |  |  |  |
| 10 |  |  |  |  |
| 10 |  |  |  |  |
| 5 |  |  |  |  |
| 13 |  |  |  |  |
| 5 |  |  |  |  |
| 34 |  |  |  |  |
| 39 |  |  |  |  |
| 5 |  |  |  |  |
| 5 |  |  |  |  |
| 5 |  |  |  |  |
| 5 |  |  |  |  |
| 5 |  |  |  |  |
| 5 |  |  |  |  |
| 5 |  |  |  |  |
| 28 |  |  |  |  |
| 5 |  |  |  |  |
| 5 |  |  |  |  |
| 5 |  |  |  |  |
| 5 |  |  |  |  |
| 5 |  |  |  |  |
| 5 |  |  |  |  |
| 5 |  |  |  |  |
| 5 |  |  |  |  |
| 5 |  |  |  |  |
| 5 |  |  |  |  |
| 5 |  |  |  |  |
| 5 |  |  |  |  |
| 5 |  |  |  |  |
| 5 |  |  |  |  |
| 12 |  |  |  |  |
| 5 |  |  |  |  |
| 5 |  |  |  |  |
| 5 |  |  |  |  |
| 5 |  |  |  |  |
| 22 |  |  |  |  |
| 5 |  |  |  |  |
| 13 |  |  |  |  |
| 18 |  |  |  |  |
| 37 |  |  |  |  |
| 5 |  |  |  |  |
| 5 |  |  |  |  |
| 5 |  |  |  |  |
| 5 |  |  |  |  |
| 5 |  |  |  |  |
| 17 |  |  |  |  |
| 5 |  |  |  |  |
| 5 |  |  |  |  |
| 11 |  |  |  |  |
| 5 |  |  |  |  |
| 5 |  |  |  |  |
| 5 |  |  |  |  |
| 5 |  |  |  |  |
| 5 |  |  |  |  |
| 5 |  |  |  |  |
| 5 |  |  |  |  |
| 5 |  |  |  |  |
| 62 |  |  |  |  |
| 5 |  |  |  |  |
| 5 |  |  |  |  |
| 41 |  |  |  |  |
| 5 |  |  |  |  |
| 5 |  |  |  |  |
| 5 |  |  |  |  |
| 5 |  |  |  |  |
| 5 |  |  |  |  |
| 5 |  |  |  |  |
| 5 |  |  |  |  |
| 5 |  |  |  |  |
| 5 |  |  |  |  |
| 5 |  |  |  |  |
| 5 |  |  |  |  |
| 5 |  |  |  |  |
| 5 |  |  |  |  |
| 5 |  |  |  |  |
| 15.9 |  |  |  |  |
| 20.1 |  |  |  |  |
| 13.4 |  |  |  |  |
| 14.3 |  |  |  |  |
| 5 |  |  |  |  |
| 19.9 |  |  |  |  |
| 12.4 |  |  |  |  |
| 5 |  |  |  |  |
| 20.2 |  |  |  |  |
| 16.5 |  |  |  |  |
| 5 |  |  |  |  |
|  |  |  |  |  |
| 15.1 |  |  |  |  |
| 20.9 |  |  |  |  |
| 19.2 |  |  |  |  |
| 17.6 |  |  |  |  |
|  |  |  |  |  |
| 13.9 |  |  |  |  |
| 15.4 |  |  |  |  |
| 13.2 |  |  |  |  |
| 5 |  |  |  |  |
| 15.8 |  |  |  |  |
| 14.3 |  |  |  |  |
| 5 |  |  |  |  |
| 15.6 |  |  |  |  |
| 12.3 |  |  |  |  |
| 5 |  |  |  |  |
| 10.7 |  |  |  |  |
| 14.9 |  |  |  |  |
| 12 |  |  |  |  |
| 5 |  |  |  |  |
| 17.2 |  |  |  |  |
| 13.8 |  |  |  |  |
| 17.2 |  |  |  |  |
| 12.7 |  |  |  |  |
| 12.2 |  |  |  |  |
| 13 |  |  |  |  |
| 5 |  |  |  |  |
| 5 |  |  |  |  |
| 12.6 |  |  |  |  |
| 5 |  |  |  |  |
| 15.9 |  |  |  |  |
| 5 |  |  |  |  |
| 5 |  |  |  |  |
| 10.6 |  |  |  |  |
| 5 |  |  |  |  |
| 5 |  |  |  |  |
| 16 |  |  |  |  |
| 34.9 |  |  |  |  |
| 5 |  |  |  |  |
| 13.6 |  |  |  |  |
| 5 |  |  |  |  |
| 5 |  |  |  |  |
| 5 |  |  |  |  |
| 5 |  |  |  |  |
| 5 |  |  |  |  |
| 23.7 |  |  |  |  |
| 22.2 |  |  |  |  |
| 16.2 |  |  |  |  |
|  |  |  |  |  |
|  |  |  |  |  |
| 12.9 |  |  |  |  |
| 5 |  |  |  |  |
| 5 |  |  |  |  |
| 12 |  |  |  |  |
| 15.2 |  |  |  |  |
| 13.2 |  |  |  |  |
| 5 |  |  |  |  |
| 11.5 |  |  |  |  |
|  |  |  |  |  |
| 37.4 |  |  |  |  |
| 22 |  |  |  |  |
| 5 |  |  |  |  |
| 5 |  |  |  |  |
| 13.6 |  |  |  |  |
| 5 |  |  |  |  |
| 5 |  |  |  |  |
| 5 |  |  |  |  |
| 15 |  |  |  |  |
| 5 |  |  |  |  |
| 17.3 |  |  |  |  |
| 23.6 |  |  |  |  |
| 5 |  |  |  |  |
| 5 |  |  |  |  |
| 5 |  |  |  |  |
| 5 |  |  |  |  |
| 14.6 |  |  |  |  |
| 5 |  |  |  |  |
| 5 |  |  |  |  |
| 5 |  |  |  |  |
| 5 |  |  |  |  |
| 5 |  |  |  |  |
| 20 |  |  |  |  |
| 5 |  |  |  |  |
| 5 |  |  |  |  |
| 15.2 |  |  |  |  |
| 5 |  |  |  |  |
| 5 |  |  |  |  |
| 35.5 |  |  |  |  |
| 5 |  |  |  |  |
